# Cost sensitive hierarchical document classification to triage PubMed abstracts for manual curation

**DOI:** 10.1186/1471-2105-12-482

**Published:** 2011-12-19

**Authors:** Emily Seymour, Rohini Damle, Alessandro Sette, Bjoern Peters

**Affiliations:** 1 The La Jolla Institute for Allergy and Immunology, 9420 Athena Circle, La Jolla, CA 92037, USA

## Abstract

**Background:**

The Immune Epitope Database (IEDB) project manually curates information from published journal articles that describe immune epitopes derived from a wide variety of organisms and associated with different diseases. In the past, abstracts of scientific articles were retrieved by broad keyword queries of PubMed, and were classified as relevant (curatable) or irrelevant (not curatable) to the scope of the database by a Naïve Bayes classifier. The curatable abstracts were subsequently manually classified into categories corresponding to different disease domains. Over the past four years, we have examined how to further improve this approach in order to enhance classification performance and to reduce the need for manual intervention.

**Results:**

Utilizing 89,884 abstracts classified by a domain expert as curatable or uncuratable, we found that a SVM classifier outperformed the previously used Naïve Bayes classifier for curatability predictions with an AUC of 0.899 and 0.854, respectively. Next, using a non-hierarchical and a hierarchical application of SVM classifiers trained on 22,833 curatable abstracts manually classified into three levels of disease specific categories we demonstrated that a hierarchical application of SVM classifiers outperformed non-hierarchical SVM classifiers for categorization. Finally, to optimize the hierarchical SVM classifiers' error profile for the curation process, cost sensitivity functions were developed to avoid serious misclassifications. We tested our design on a benchmark dataset of 1,388 references and achieved an overall category prediction accuracy of 94.4%, 93.9%, and 82.1% at the three levels of categorization, respectively.

**Conclusions:**

A hierarchical application of SVM algorithms with cost sensitive output weighting enabled high quality reference classification with few serious misclassifications. This enabled us to significantly reduce the manual component of abstract categorization. Our findings are relevant to other databases that are developing their own document classifier schema and the datasets we make available provide large scale real-life benchmark sets for method developers.

## Background

The Immune Epitope Database and Analysis Resource (IEDB, http://www.iedb.org) contains epitope information and analysis tools [1,2]. Scientific articles and direct submissions from researchers provide the content from which IEDB curators manually extract epitope related information and enter it into the database [3]. The database is freely available to the scientific community.

**Figure 1:**
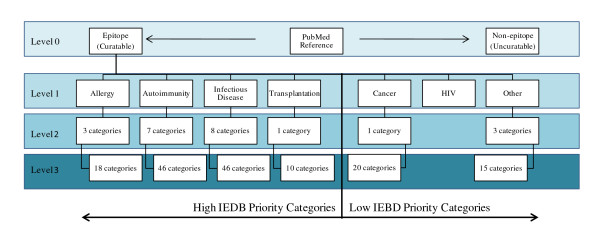
**IEDB PubMed abstract triaging process**. Abstracts of references retrieved from the PubMed queries that have not been introduced to the IEDB's database and curation pipeline proceed to at least one of four hierarchical levels of classification. At Level 0, an abstract is evaluated for epitope-specific content. Abstracts which contain epitope-specific data are assigned to one of the seven Level 1 categories. References receive increasingly specific category assignments at Levels 2 and 3. High IEDB priority categories are Allergy, Autoimmunity, Infectious Disease, and Transplantation. Low IEDB priority categories are Cancer, HIV, and Other. Transplant and Cancer references are not assigned Level 2 categories. HIV references do not receive Level 2 or 3 category assignments.

The IEDB journal article triaging process goes as follows. Four times each year a query is run containing multiple epitope-specific keywords and logical operators [2,4] to identify new references for curation in PubMed. The abstracts of references that have not been previously introduced to the IEDB's internal database ("new references") are evaluated and hierarchically classified (Figure 1). Relevant references must contain epitope-specific data and an epitope structure [3]. Irrelevant, or uncuratable, abstracts are entered into the IEBD's internal database but are not further processed (Level 0 in Figure 1). Next, each article containing epitope information is categorized into one of seven Level 1 categories, namely Allergy, Autoimmunity, Infectious Disease, Transplantation, Cancer, HIV, and "Other" (Level 1 in Figure 1). References in the Other category do not meet the criteria for placement into the remaining six categories yet contain relevant epitope information [5]. Curation priorities of the IEDB, established by The National Institute of Allergy and Infectious Diseases (NIAID), are references in the Allergy, Autoimmunity, Infectious Disease, and Transplantation categories. Level 2 classification assigns each reference to a more specific category (Level 2 in Figure 1). An autoimmune reference, for example, may be categorized into the Beta-Amyloid, Diabetes, General Autoimmune, Lupus, Multiple Sclerosis, Myasthenia Gravis, or Rheumatoid Arthritis category. The final level of classification breaks these down further (Level 3 in Figure 1). For example, Diabetes references may be assigned to one of seven Level 3 categories: Glutamic Acid Decarboxylase, Heat Shock Proteins, Insulinoma-Associated Protein-2, Islet-Specific Glucose-6-Phosphatase Catalytic Subunit-Related Protein, Insulin/Proinsulin, Other, or Various/Multiple for abstracts that refer to several Diabetes categories. The Level 1-3 categorizations of the references in the IEDB, first presented in [5], are in additional file 1.

We have previously described our implementation of a Naïve Bayes classifier to automate the Level 0 classification of curatable vs. uncuratable references [4]. The additional categorization of curatable references into disease specific subsets was added more recently and performed manually. The main goal of the present study was to develop and implement classifiers to reduce the amount of time required for an article to proceed from the query to curation and maintain consistency in the criteria used to evaluate the references.

There are two approaches to classify references into a hierarchical categorization scheme. Either, references are assigned the final category in a single step, or classification is done stepwise, deciding at each level which category of several distinct siblings is the most appropriate. The latter process, classification that occurs in several stages, involves the construction and implementation of hierarchical classifiers [6-11]. Hierarchical classification permits increased specificity in feature selection because classification is conducted on small groups of related references instead of in one step among all references in a dataset [12]. Dumais and Chen [13] implemented a hierarchical SVM classification system to classify a set of pages from LookSmart. Hierarchical SVM classifiers based on the support vector clustering method for automatic document classification resulted in improved classification accuracy compared to the k-NN and decision tree systems [12].

The IEDB has processed a large dataset of 89,884 references classified by a human expert. Torii and Liu [14] built an ensemble of SVM classifiers and compared their performance to multinomial Naïve Bayes and single SVM classifiers using several published datasets, including a dataset from the IEDB [4]. When applied to references in the IEDB dataset [4], the ensemble of SVM classifiers outperformed Naïve Bayes and single SVM classifiers [14]. We therefore implemented and compared Naïve Bayes and SVM classifiers for performance on discriminating between curatable and uncuratable references in our dataset. SVM non-hierarchical classifiers and a hierarchical application of SVM classifiers were subsequently built and compared for performance on predicting Level 1-3 category assignments. Using the output scores from the hierarchical application of SVM classifiers, neural network classifiers assigned Level 1-3 categories to each reference. Finally, cost sensitivity was incorporated into the design of the hierarchical application of SVM classifiers to minimize misclassifications of priority references. We tested our design on an independent dataset of 1,388 references. Here we report our results which highlight the superior performance of the cost sensitive hierarchical application of SVM classifiers as applied to the reference evaluation process in the IEDB. For the purposes of the work performed in this paper, any use of the term "hierarchical SVM" refers to our system which used a hierarchical application of SVM classifiers.

## Results

### Naïve Bayes and Support Vector Machine classifier training for Level 0

The first step in the curation of references retrieved by automatic queries of the PubMed library is to determine whether or not a reference is relevant to the scope of the IEDB database. We previously implemented a Naïve Bayes classifier to automate this step, referred herein as "Level 0" [4]. Based on a report [14] that SVM classifiers can outperform Naïve Bayes classifiers on our published dataset [4], we compared the performance of Naïve Bayes and SVM classifiers for the IEDB's document classification purposes. For the curatability prediction we wanted to maintain a false negative rate of less than 5%, a value that corresponds to the inherent disagreement rate for an abstract scan between two human experts [4]. At a false negative rate of 5% or less, we then wanted to maximize the true positive rate. We adapted the SVM code in [14] into python scripts and used 22,274 curatable (positive examples) and 67,610 uncuratable (negative examples) abstracts previously classified by a human expert to develop a SVM training algorithm to build a set of models to automate Level 0.

**Figure 2:**
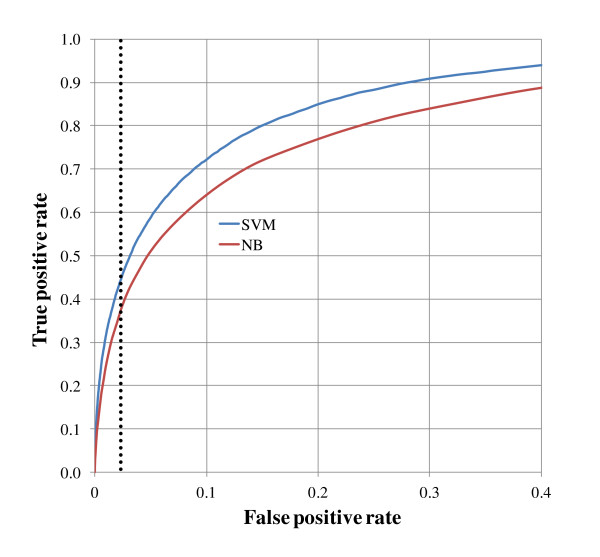
**Comparison of Naïve Bayes and SVM algorithms at training Level 0**. The performance of the Naïve Bayes and SVM classifiers was evaluated with 10-fold cross-validation. As is shown in the ROC curve, the SVM classifier outperformed the Naïve Bayes classifier on curatability predictions for the cross-validation dataset of 89,884 abstracts. The AUC value for the SVM classifier was 0.899 and the AUC value for the Naïve Bayes classifier was 0.854. At the 5% false negative rate for the curatability decision, the SVM classifier had a true positive rate of 41.4% and the Naïve Bayes classifier had a true positive rate of 33.5%.

We evaluated the performance of the Naïve Bayes and SVM classifiers with 10-fold cross-validation and used the Area Under the Curve (AUC) values to compare performance (Figure 2). An AUC value of 0.899 was obtained for the SVM classifier compared to a Naïve Bayes AUC value of 0.854. At a false negative rate of 5% the true positive rate for the SVM classifier was 41.4% and 33.5% for the Naïve Bayes classifier. Based on these results we transitioned from a Naïve Bayes to SVM classifier for all subsequent applications.

### Support Vector Machine classifier training for subsequent levels

Next, curatable abstracts are assigned to one of seven Level 1 categories: Allergy, Autoimmunity, Infectious Disease, Transplantation, Cancer, HIV, or Other. In order to automate the Level 1 assignments a training dataset of 22,833 abstracts assigned to one of the seven Level 1 categories by a human expert was used to build seven SVM classifiers. The classifiers were trained such that all abstracts from a single category received a "yes" and the remainder of the abstracts received a "no." For example, the Autoimmunity training set had "yes" for 4,350 abstracts and "no" for the remaining 18,483 abstracts. The seven Level 1 classifiers underwent 10-fold cross-validation and, as shown in Table 1, all seven classifiers consistently achieved AUC values above 0.98.

**Table 1:** Hierarchical SVM classifier performance on training dataset for Level 1 predictions.

Level 1 category	Curatable abstracts	AUC individual category (SVM)	Category prediction accuracy (%)(MLP)
Allergy	1146	0.994	91.6
Autoimmunity	4350	0.988	88.9
Infectious Disease	7525	0.989	92.7
Transplantation	888	0.985	76.4
HIV	2369	0.989	92.6
Cancer	2650	0.988	89.8
Other	3905	0.985	85.4
**Total**	**22833**		**89.7**

Performance was evaluated with 10-fold cross-validation. An AUC value was calculated for a given category, where documents assigned by the expert to be of that category are considered "positive" while documents assigned to any other category are negative. To evaluate the performance of the document classification by the Multilayer Perceptron (MLP) into specific categories, we calculated the percent agreement of categories. The AUC and category prediction accuracy values are entered for each Level 1 category in addition to the total prediction accuracy.

We tested different algorithms in WEKA [15] to find the optimal function that takes as an input the scores from the seven SVM classifiers and returns as an output the Level 1 category assignments. We tested these algorithms in the same cross-validation setup used to evaluate the individual SVM classifiers. To evaluate the performance of this multi-category classification problem, we cannot use AUC values that we use for the individual category SVM classifiers. Instead, we compare the accuracy of classification in each category. The Multilayer Perceptron algorithm [16] returned the strongest results, predicting correct Level 1 categories for 89.7% of the references. Furthermore, it correctly predicted 90.3% of the references falling into high priority Level 1 categories. It was therefore decided to implement the Multilayer Perceptron algorithm into our design at Levels 1-3.

In Level 2 abstracts from each Level 1 category are further assigned to finer categories. The Autoimmunity category, for example, contained 4,350 curatable training abstracts assigned to one of seven finer categories: Beta-Amyloid, Diabetes, General Autoimmune, Lupus, Multiple Sclerosis, Myasthenia Gravis, and Rheumatoid Arthritis. SVM classifiers were trained only on the abstracts that had been assigned into the same Level 1 category. For example, training data for the Diabetes classifier consisted of positive examples for Diabetes references and negative examples containing all remaining abstracts categorized as autoimmune references. The Level 2 classifiers underwent five-fold cross-validation and yielded satisfactory AUC and category prediction accuracy values which are displayed in Table 2 for the Autoimmunity Level 2 categories. AUC values above 0.98 were consistently achieved.

**Table 2:** Hierarchical SVM classifier performance on Autoimmunity training dataset for Level 2 predictions.

Level 2 category	Curatable abstracts	AUC individual category (SVM)	Category prediction accuracy (%)(MLP)
Beta-Amyloid	213	.998	97.2
Diabetes	443	.998	96.6
General Autoimmune	1311	.987	87.6
Lupus	704	.988	94.6
Multiple Sclerosis	957	.990	97.3
Myasthenia Gravis	221	.992	95.0
Rheumatoid Arthritis	501	.990	84.0
**Total**	**4350**		**92.2**

Performance was evaluated with five-fold cross-validation. The AUC and category prediction accuracy values are entered for each Autoimmunity Level 2 category.

Finally, SVM models were trained to assign Level 3 categories to abstracts. One hundred fifty-five classifiers were designed for Level 3. For example, abstracts placed into the Autoimmunity category (Level 1) and assigned to the Diabetes category (Level 2) received an assignment to the Glutamic Acid Decarboxylase (GAD), Heat Shock Proteins (HSP), Insulinoma-Associated Protein-2 (IA2), Islet-Specific Glucose-6-Phosphatase Catalytic Subunit-Related Protein (IGRP), Insulin/Proinsulin (INSULIN), Other (OTH), or Various/Multiple (VAR) Level 3 category. SVM classifiers were trained only on the abstracts that had been assigned into the same Level 2 category. For example, training data for the Heat Shock Proteins classifier consisted of positive examples for Heat Shock Protein references and negative examples containing all remaining abstracts categorized as Diabetes references. In Table 3 we present the AUC and category prediction accuracy values for the Diabetes Level 3 category classifiers. Prediction performance for the OTH and VAR categories was much lower (AUC of .823 and .695, respectively) than the remaining categories (above 0.96). This reflects that references in those categories are much more heterogeneous.

**Table 3:** Hierarchical SVM classifier performance on Diabetes training dataset for Level 3 predictions.

Level 3 category	Curatable abstracts	AUC individual category (SVM)	Category prediction accuracy (%)(MLP)
GAD	148	.966	87.2
HSP	19	.997	89.5
IA2	26	.987	80.8
IGRP	16	.995	75.0
INSULIN	126	.964	87.3
OTH	79	.823	53.2
VAR	29	.695	13.8
**Total**	**443**		**75.6**

The AUC and category prediction accuracy values are entered for each Diabetes Level 3 category.

### Performance comparison between a non-hierarchical and a hierarchical application of SVM classifiers

Having trained the hierarchical application of SVM classifiers to predict categories we compared their performance to non-hierarchical classifiers. The construction of 156 non-hierarchical SVM classifiers would have required a substantial amount of time and computer power so we limited the comparison to the Autoimmunity category to build non-hierarchical SVM classifiers and compare their performance against a hierarchical application of Autoimmunity SVM category classifiers. For example, to train the non-hierarchical SVM classifiers to predict Diabetes abstracts into the Diabetes category we used the 443 Diabetes training abstracts as positive examples against the remainder of the 22,390 curatable abstracts (negative examples). The hierarchical application of SVM classifiers for the Diabetes references were trained with the 443 Diabetes abstracts (positive examples) against the remainder of the 3,907 curatable Autoimmunity abstracts (negative examples). Training time for the hierarchical classification approach is much shorter because only abstracts within the same category are considered. Furthermore, positive and negative training examples were less imbalanced in the hierarchical scheme. The performance of hierarchical versus non-hierarchical classifiers was compared using the same cross-validation set up as before. Performance was compared separately for each of the seven Level 2 categories under Autoimmunity. AUC values were used for each category to evaluate the ability of the classifier to distinguish documents belonging to that category from documents belonging to any of the other six categories. The AUC values for the Autoimmunity non-hierarchical and hierarchical application of SVM classifiers are shown in Table 4. The average AUC value for the non-hierarchical classifiers was 0.983 and the average AUC value for the hierarchical application of classifiers was 0.992. This difference is significant with a p-value of .009 (paired t-test, 2-sided). Based on these results the hierarchical application of SVM classifiers are not only faster to train but also outperformed non-hierarchical classifiers.

**Table 4:** Performance comparison of non-hierarchical and hierarchical SVM classifiers.

Category	Number of curatable abstracts	Non-hierarchical AUC	Hierarchical AUC
Beta-Amyloid	213	.994	.998
Diabetes	443	.997	.998
General Autoimmune	1311	.967	.987
Lupus	704	.975	.988
Multiple Sclerosis	957	.982	.990
Myasthenia Gravis	221	.983	.992
Rheumatoid Arthritis	501	.983	.990

AUC values from the non-hierarchical and hierarchical Autoimmunity category SVM classifiers.

### Implementation of cost sensitive matrices

The IEDB was funded to curate Allergy, Autoimmunity, Infectious Disease, and Transplantation references, which makes HIV, Cancer, and Other references a low priority for our curation. There is a substantial cost associated with misclassifying a high priority reference into a low priority category since abstracts placed in the low priority categories undergo no further review by a human expert and are not curated. Thus these misclassifications result in missed high priority references. To reduce the number of high priority references misclassified into the low priority categories, cost sensitive classification was implemented by specifying cost matrices for the category selection step performed by the Multilayer Perceptron neural network. There were seven categories in Level 1, so we built a 7 × 7 cost matrix (see additional file 2) with a cost of zero for all correct category assignments; a cost of 0.2 for an abstract that was predicted into a low priority category and the human expert identified a different low priority category (for example, the classifier predicted Cancer whereas the human expert designated the abstract as HIV); a cost of one in the instance that the classifier and human expert placed an abstract into one of the four high priority categories but the human expert overruled the classifier's category prediction (for example, the classifier predicted Autoimmunity whereas the human expert placed the abstract into Allergy); and a cost of five for an abstract that the human expert placed into a high priority category but the classifier predicted into a low priority category (for example, the classifier predicted Cancer whereas the human expert placed the abstract into Autoimmunity). The Multilayer Perceptron is trained to minimize total cost, and will therefore specifically avoid placing high priority abstracts into a low priority category.

Using the 22,833 curatable training abstracts we compared outcomes for the Level 1 category assignments with or without cost sensitivity (Table 5). With the implementation of cost sensitivity at Level 1 there was a decrease in the number of high priority references misclassified into low priority categories, from 987 to 467, as desired by our curation process. As expected, at the same time the number of references incorrectly classified as high priority went up from 1,207 to 2,042. Essentially, the classifiers will now rather assign a borderline reference into a high priority category, which is exactly what we wanted to achieve.

**Table 5:** Comparison of training Level 1 category predictions with and without cost sensitivity.

Number of references	No cost	Cost sensitive
**Classified as high priority**	**13722**	**15020**
Correct classification	12515	12978
Incorrect, should be...	1207	2042
Other high priority	407	464
Low priority	800	1578
**Classified as low priority**	**9111**	**7813**
Correct classification	7799	7112
Incorrect, should be...	1312	701
Other low priority	325	234
High priority	987	467

The number of references predicted into the Level 1 categories with and without cost sensitivity. In the cost sensitive scenario, there was a decrease in the number of high priority references misclassified into low priority categories.

Cost sensitivity was applied in Level 2 or 3 classification when "Other" was present as a category. This was implemented in order to reduce incorrect predictions into this category and maximize predictions into the more specific categories. As an example, the cost matrices at Level 3 for Diabetes references are shown in additional file 3. In Table 6 we compare the results of applying no cost and cost sensitive SVM classifiers for the Diabetes Level 3 category assignments. With the implementation of cost sensitivity, fewer references were predicted into the Other category. Overall category prediction accuracy decreased but category prediction accuracy into the well-defined categories improved with the cost sensitive SVM classifiers.

**Table 6:** Comparison of no cost and cost sensitive classification on training Level 3 Diabetes references.

Level 3 categories	Curatable abstracts	Correct predictions with no cost	No cost category prediction accuracy (%)	Correct predictions with cost sensitivity	Cost sensitivity category prediction accuracy (%)
GAD	148	129	87.2	140	94.6
HSP	19	17	89.5	18	94.7
IA2	26	21	80.8	21	80.8
IGRP	16	12	75.0	13	81.3
INSULIN	126	110	87.3	120	95.2
OTH	79	42	53.2	9	11.4
VAR	29	4	13.8	3	10.3
**Total**	**443**	**335**	**75.6**	**324**	**73.1**

The number of correct predictions and category prediction accuracy for no cost and cost sensitive classification of training Level 3 Diabetes references.

### Testing performance of cost sensitive hierarchical SVM classifiers on an independent benchmark dataset

Using the methodology identified as optimal in cross-validation in the previous sections, we tested the performance of our approach on an independent dataset of 1,388 abstracts retrieved on September 20, 2009 that were not part of the cross-validation datasets. The SVM based main classifier (Level 0) predicted that 642 of the 1,388 references were curatable using the previously determined cutoff aimed at achieving 95% sensitivity. A human expert evaluated the classifier's performance and confirmed that 287 of the 642 references were curatable. Of the 746 references predicted to be uncuratable, the human expert identified 14 that were indeed curatable. That corresponds to a sensitivity of 95.3% with a specificity of 67.3% which is in our desired range. These results reflect the thresholds purposely set to maximize sensitivity in order to avoid discarding curatable references.

We compared the classifier's predictions for the 287 curatable abstracts against the human expert's assignments. Table 7 shows a matrix of the classifier's category predictions and human expert's assignments for the 287 abstracts confirmed as curatable. The classifier correctly predicted 271 (94.4%) of the Level 1 category assignments. Of the 287 curatable abstracts, 186 were predicted into high priority categories by the classifier. The human expert assigned 184 of the abstracts to high priority categories and of those, confirmed that 96.2% of the classifier's high priority category predictions were correct. Of the 101 references predicted into low priority categories, only four references were classified as high priority categories by the human expert. Three references predicted into low priority categories were reassigned to different low priority categories.

**Table 7:** Comparison of predicted and actual Level 1 category assignments on independent dataset.

Classifier								
**Human Expert**		**Allergy**	**Autoimmunity**	**Infectious Disease**	**Transplantation**	**Cancer**	**HIV**	**Other**
	Allergy	**11**	1	0	0	0	0	0
	Autoimmunity	0	**58**	0	0	0	0	1
	Infectious	0	1	**100**	0	1	0	2
	Disease							
	Transplantation	0	1	0	**8**	0	0	0
	Cancer	0	1	0	1	**41**	0	2
	HIV	0	0	2	0	0	**33**	0
	Other	0	1	1	0	1	0	**20**
	**Total**	**0**	**5**	**3**	**1**	**2**	**0**	**5**
	**Incorrect:16**							
	**Uncuratable**	**17**	**62**	**120**	**26**	**68**	**45**	**17**

Columns represent predictions by the classifier and rows represent the Level 1 category assigned by a human expert. For example, one reference predicted as Transplant was actually Cancer. The Total Incorrect row represents the total number of references that were predicted into Level 1 categories by the classifier that differed from the decision of the human expert. Of the 642 abstracts predicted to be curatable, 355 abstracts were overruled as uncuratable which can be seen in the Uncuratable row. Of the 287 curatable abstracts, 94.4% were assigned to the correct Level 1 category.

Next, we compared the Level 2 and 3 assignments of the human expert with the hierarchical classifier system (Table 8) for the 287 curatable abstracts. This shows, for example, that the human expert placed 59 of the curatable abstracts into the Level 1 Autoimmunity category. The Autoimmunity classifiers predicted the correct category for 58 of the curatable abstracts at Level 2 and 48 of the curatable abstracts at Level 3. The percent of correct predictions for the high priority categories at Levels 1-3 (96.2%, 95.4%, and 84.8%, respectively) exceeded those for the low priority categories at Levels 1-3 (91.3%,82.6%, and 75.0%, respectively) as desired based on our cost assignments.

**Table 8:** Performance of the hierarchical SVM classifiers at Levels 2 and 3.

Level 1	Assigned by expert	Level 2: Correct predictions	Level 2: Correct predictions (%)	Level 3: Correct predictions	Level 3: Correct predictions (%)
Allergy	12	11	91.7%	9	75.0%
Autoimmunity	59	58	98.3%	48	81.4%
Infectious Disease	104	98	94.2%	91	87.5%
Transplantation	9	n/a	n/a	8	88.9%
**Subtotal**	**184**	**167**	**95.4%**	**156**	**84.8%**
Cancer	45	n/a	n/a	32	71.1%
HIV	35	n/a	n/a	n/a	n/a
Other	23	19	82.6%	19	82.6%
**Subtotal**	**103**	**19**	**82.6%**	**51**	**75.0%**
**Total**	**287**	**186**	**93.9%**	**207**	**82.1%**

Transplantation and Cancer do not receive Level 2 category assignments. HIV does not receive Level 2 or 3 category assignments.

For benchmarking purposes, we are making the entire cross-validation and independent datasets available as additional files (see additional files 4, 5, and 6).

## Discussion

Here we present a practical application of automated document classification for the purposes of the IEDB. This was prompted by the desire to increase efficiency in the review process of the several thousand abstracts retrieved from querying PubMed each year. The abstract review process assesses relevancy to the database and places curatable abstracts into a disease-specific category. We automated the assignment of categories to make this a more efficient process. In this process, we tested different methodologies and tools, and believe that our results should prove useful to researchers working on similar tasks.

In the past, we used a Naïve Bayes classifier to predict curatability [4]. SVM classifiers were reported to outperform other classifiers [14,17-19] and one group [14] showed high SVM performance on our previously published dataset [4]. We compared performance between Naïve Bayes and SVM classifiers and confirmed that SVM outperformed Naïve Bayes classifiers when distinguishing between curatable and uncuratable abstracts. In our original publication [4] we also attempted to use SVM classifiers but achieved much poorer performance, most likely due to sub-optimal choice of parameters. After our present extensive tests, we conclude that SVM classifiers are overall superior to Naïve Bayes classifiers for our abstract classification task.

We also compared the performance of a non-hierarchical and a hierarchical application of SVM classifiers in order to determine the best approach for automating the disease category assignments. Based on the higher AUC values achieved using the hierarchical application of SVM classifiers we adopted the hierarchical strategy for classifying the abstracts. Our results confirm previous findings [20-24] that at least if there is a sufficiently large base of data, hierarchical classifiers perform better. We believe that this is primarily due to the higher homogeneity of the abstracts encountered when making category assignments, which will improve the ability to reliably make finer distinctions between related categories.

Cost-sensitive classification had a major positive impact on the practical performance of our predictions. As all references predicted to be in high priority categories will be manually reviewed as part of the curation process, it was most important for us to ensure that few high priority references were misclassified as being low priority. We accomplished this by simply assigning different costs to the errors made by the Multilayer Perceptron that assigns categories based on the SVM output scores. A similar approach was taken by Cai and Hofmann [22] when they implemented cost sensitive document categorization with hierarchical SVM classifiers on the WIPO-alpha collection and included interclass relationships.

The ability to not only identify relevant references, but also group them into related subject areas, has benefits for curators and management. Grouping articles enables coordinated curation of related content, the prioritization of particular subject areas over others, and the assignment of specific curators to categories that require certain expertise. Management can account for progress on particular subject areas and can re-direct effort to priority references. For example, in light of the 2009 H1N1 pandemic, the IEDB re-directed curation priorities to immediately curate all influenza related articles [25]. This was eased by the ability to quickly identify the relevant articles based on their available categorization.

The cross-validation and independent test datasets we compiled are made available as additional files (see additional files 4, 5, and 6). We strongly encourage the use of this corpus for benchmarking purposes, as has been done with our previous published dataset [2,5,14,26-37]. Our dataset has been carefully manually inspected. All abstracts were reviewed by a senior immunologist and for those abstracts deemed curatable, the full text reference was retrieved and reviewed in detail by an IEDB curator. The size of our dataset, the application of hierarchical classification, and the expert assignments make this a unique and practically relevant corpus of data for biomedical text categorization.

## Conclusions

Since the inception of the IEBD, over 100,000 abstracts have been evaluated for curatability. A human expert requires constant time to evaluate thousands of abstracts while an automated classifier can learn from past decisions and will surpass the expert in speed. Automating the categorization of documents enabled us to expedite the preparation of documents for curation and coordinate curation efforts, both of which are time efficient and cost effective approaches. We also took into consideration our curation priorities and implemented cost sensitivity to reduce the possibility that high priority abstracts were misclassified. Our datasets and methods may be relevant to other database and prediction methods developers with similar goals.

## Methods

### Classifiers

The Naïve Bayes classifier was based upon the algorithm in [4] and implemented using python scripts. SVM classifiers were constructed using the SVM Light code [14,38] and adapted to python scripts.

### Feature selection

Titles and abstracts from PubMed were parsed and NCBI stopwords [39] and rare words occurring in less than three documents were excluded. We applied the algorithms for the inverse of document frequency (IDF) and information gain (IG) as well as the feature vector generation methods used in [14]. Specifically, documents were represented in a vector format and each value in a vector associated with a feature word is the frequency of that feature word in the document (TF) weighted by the inverse of the document frequency (IDF) [14]. Feature words were selected by applying an information gain threshold of 100.

### Classifier ensemble

For each classification task using SVMs, we built a classifier ensemble to improve the robustness of the classification similar to what was done in [14]. To construct the ensemble, the training set was split into ten disjoint subsets, and ten classifiers were trained leaving out one of the ten subsets from the training data in each case. In contrast to [14], we used the same information gain cutoff value of 100 for each of these SVM classifiers. When making predictions on a blind set, each PMID received ten prediction values and the values were averaged to assign a final prediction value to the PMID.

### Multilayer Perceptron algorithm

A Multilayer Perceptron (MLP) is feed-forward neural network consisting of an input layer, one or more hidden layers, and an output layer that is used to model non-linear functions [40,41]. We used the Multilayer Perceptron implemented in the WEKA [16] package to make a category assignment to a PMID based on SVM classifier scores for each of the available categories. When applied to our work at Level 1, for example, the scores from the seven Level 1 SVM classifier ensembles were input to the Multilayer Perceptron in order to make a single Level 1 category assignment. The WEKA default parameters were used during training, which included setting the number of hidden layers equivalent to the number of available categories divided by two.

### Performance measures

Ten-fold cross-validation [42] was conducted to evaluate classifier performance at Levels 0 and 1. Five-fold cross-validation was conducted to evaluate classifier performance at Level 2. In order to carry out ten-fold cross-validation for classifier training at Level 0, for example, the 89,884 references in the training dataset were divided into ten subsets. Each of the subsets was used as a test set once and the remaining nine sets of references were used as the training set. This was repeated ten times until the tenth subset was used as the test set and subsets 1-9 comprised the training set.

Area Under the Curve (AUC) values [43-45] were used to evaluate performance of the SVM classifiers for individual categories. An AUC value represents the likelihood that a classifier correctly gave a higher prediction score to a positive instance (belonging to a category) above a negative instance (not belonging to the category) [43-45]. During cross-validation, the classifiers and feature files built from the training sets were used to compute prediction scores for PMIDs in the blind sets for a given category. For a given cutoff value for the prediction score, PMIDs are separated by their scores into those predicted to belong to a category and those predicted not to belong to a category. Two variables are calculated for a given cutoff: true positive rate (true positives/total positives) and false positive rate (false positives/total negatives). By systematically varying the cutoff from the lowest to the highest predicted score, a ROC curve such as Figure 2 is generated. Prediction performance is measured by the AUC, which is 0.5 for random predictions and 1.0 for perfect predictions. To evaluate the performance of the document classification by the Multilayer Perceptron into specific categories, we calculated the percent agreement of categories instead. A paired, two-sided t-test was used to generate a p-value to compare the average AUC values for the non-hierarchical and hierarchical application of SVM Autoimmunity classifiers.

### Classifier training and operating time

The computer system used was running Ubuntu 10.4 with Intel ^® ^Core 2 Quad processors. Classifier training required 48 hours. Predictions were generated in two to five hours, depending on the classification method and size of the dataset.

## Authors' contributions

ES drafted the manuscript and helped with data collection. RD carried out classifier training, testing, data collection, and helped to draft the manuscript. AS supervised and helped to design and draft the manuscript. BP designed the study, evaluated the approaches, and helped to design and draft the manuscript. All authors read and approved the final manuscript. The authors do not have any competing interests.

## Supplementary Material

Additional file 1 **Level 1-3 categorizations of references in the IEDB**. The tables show the Level 1-3 categorizations of the references in the IEDB, first presented in [5]. Click here for file

Additional file 2 **Level 1 uniform cost and cost sensitivity matrices**. The uniform cost and cost sensitivity matrices at Level 1. Click here for file

Additional file 3 **Diabetes Level 3 uniform and cost sensitivity matrices**. Uniform cost and cost sensitivity values for Diabetes references at Level 3. Cost sensitivity was implemented when Other was present as a category. Columns represent predictions by the classifier and rows represent the Level 3 category assigned by a human expert. The last row ("Total Incorrect") represents the number of references in which the classifier's prediction was overruled by the human expert. With the implementation of cost sensitivity, fewer references were predicted into the Other category. Click here for file

Additional file 4 **Level 0 cross-validation dataset**. The cross-validation dataset for Level 0 was used to develop a SVM training algorithm to build a set of models to automate the Level 0 decision. A human expert manually classified 89,884 abstracts retrieved from PubMed as "curatable" or "uncuratable." Of the 89,884 abstracts, 22,274 are curatable and 67,610 are uncuratable. Additional file 4 contains the PubMed Identification Numbers of the 89,884 abstracts and Level 0 categorizations. Click here for file

Additional file 5 **Levels 1-3 cross-validation dataset**. The cross-validation dataset for Levels 1-3 was used to build SVM classifiers to automate the assignment of Level 1-3 categories to curatable references. Additional file 5 contains the PubMed Identification Numbers and Level 1-3 categorizations for the 22,833 curatable abstracts manually assigned Level 1-3 categories by a human expert. Click here for file

Additional file 6 **Independent test dataset**. At Level 0, the SVM classifier predicted that 647 of the 1,388 references from the independent test dataset were curatable. The human expert confirmed that 287 of the 647 references were curatable. Additional file 6 contains the PubMed Identification Numbers, predicted Level 1-3 categorizations, and the expert's Level 1-3 categorizations for the 287 curatable references. Click here for file
