# Supplementary material for: Cost sensitive hierarchical document classification to triage PubMed abstracts for manual curation
Source: BMC Bioinformatics. 2011 Dec 19;12:482. doi: 10.1186/1471-2105-12-482 (PMC3314711; doi:10.1186/1471-2105-12-482)
Supplement: Additional file 1 — Level 1-3 categorizations of references in the IEDB. The tables show the Level 1-3 categorizations of the references in the IEDB, first presented in [5]. [file 1471-2105-12-482-S1.PDF]

Categorization of Allergy references

**Level 1**	**Level 2**	**Level 3**	**Abbreviation**
Allergy			
	Plant		
		Beulaceae (Birch Family)	BET
		Cupressaceae (Cypress, Cedar Family)	CED
		Other Trees	OTTREES
		Fabaceae (Peas, Soybean, Peanut Family)	ARA
		Latex (Hevea)	LAT
		Other Flowering Plants	OTPLA
		Timothy Grass, Phl, Poaceae	GRA
		Gluten, Coeliac Disease	GLUT
	Eukaryota (Non-Plant)		
		Insecta	INS
		Arachnida (Acari, Mites, and Ticks)	DER
		Mammals	MAM
		Birds (Aves)	BIRDS
		Other Bilateria (Roundworms, Nematodes, Schistosoma, Parasites, Crustacea)	WORM
		Fungi	FUN
	Other Allergen		
		DNP, DNFB, TNP, TNCB, DNCB	DNP
		Other Haptens	OTAP
		Metals (nickel, berillium, and others)	MET
		Other Allergens	OTH

Allergy references are categorized based on the source of the allergen. There are three Level 2 categories and 18 Level 3 categories.

Categorization of Autoimmunity references

Level 1	Level 2	Level 3	Abbreviation
Autoimmunity			
	Rheumatoid Arthritis		
		Citrullinated Auto-antibodies	CCP
		Collagen (Type II)	CII
		Collagen, Type II (260-267; 250-270)	C260
		Heat Shock Proteins	HSP
		Human Cartilage	HC
		RA Motif/Shared Epitope	RAM
		Rheumatoid Factors	RF
		Other (Various/Multiple/Other Subtypes)	OTH
	Diabetes		
		Glutamic Acid Decarboxylase	GAD
		Heat Shock Proteins	HSP
		Insulin/Proinsulin	INSULIN
		Insulinoma-Associated Protein-2	IA2
		Islet-Specific Glucose-6-Phosphatase Catalytic Subunit-Related Protein	IGRP
		Various/Multiple Subtypes	VAR
		Other (Undefined Subtype)	OTH
	Multiple Sclerosis		
		Anti-Myelin Oligodendrocyte Glycoprotein	MOG
		Anti-Myelin Oligodendrocyte Glycoprotein (37-48; 35-55; 42-53)	MOG35
		General Myelin Basic Proteins	MBP
		General Myelin Basic Proteins (78-99; 83-99; 85-99; 83-92; 87-99)	MBP78
		General Myelin Basic Proteins Ac 1-9; 1-10	Ac1-9
		Proteolipid Protein	PLP
		Proteolipid Protein 139-151	P139
		T-cell Receptor Peptides	TCR
		Various/Multiple Subtypes	VAR
		Other (Undefined Subtype)	OTH
	Lupus		
		Antibodies	ABS
		Antiphospholipid/Cardiolipin	APL
		DNA, Nucleic Acids	DNA
		Histones (H1, H4, H3, H2A, H2B)	HIS
		Inositol 1,4,5-Triphosphate Receptors/NO/Sulfatide Antigens	APT
		La/SSB	SSB
		Ribosomal P Proteins (Rib-P)	RIP
		Ro/SSA	SSA
		Sm Autoantigen/Small RNP/RNPA	SM
		Other (Various/Multiple/Other Subtypes)	OTH
	General Autoimmune		
		Eppin/Luteinizing Hormone/Human Chorionic Gonadotropin/Pellucida	CONTRA
		Experimental Autoimmune Myocarditis	EAM
		Human Interphotoreceptor Retinoid-Binding Protein (IRBP)/Uveitis	IRBP

		Interferons	IFN
		Liver	LIV
		Pemphigus/Desmoglein	PEMP
		Thyroid (Graves' Disease)	TYR
		Von Willebrand/Haemophilia	HAEM
		Other (Undefined Subtype)	OTH
	Myasthenia Gravis	Myasthenia Gravis/Torpedo Acetylcholine Receptor	MYA
	Beta-Amyloid	Beta-Amyloid	BETAAM

Autoimmunity references are categorized according to the particular autoimmune manifestation. There are seven Level 2 categories and 46 Level 3 categories.

Categorization of Infectious Disease references

Level 1	Level 2	Level 3	Abbreviation
Infectious Disease			
	ssRNA (-) Strand Virus		
		Paramyxoviridae (Respiratory Syncytial Virus, Measles, Mumps)	PARAV
		Rhabdoviridae (Vesicular Stomatitis Virus, Rabies)	VSV
		Other Mononegavirales (Ebola, Borna)	OTMON
		H3N2 Subtype Influenza A	H3N2
		H1N1 Subtype Influenza A	H1N1
		Other Influenza A Subtypes (not H3N2, H1N1)	OTFLU
		Other Orthomyxoviridae (Unclassified orthomyxoviridae, Influenza B/C)	OTORTHOM
		Other ssRNA (-) Strand Viruses (Rift Valley Fever Virus, Hanta, Arenavirus)	OTssRNA-
	ssRNA (+) Strand Virus		
		Hepatitis C Virus	HCV
		Dengue Virus	DENG
		Flaviviridae (Pestivirus, West Nile Virus, Yellow Fever, Tick-Borne Encephalitis, Unclassified Flaviviridae, Other Encephalitis)	OTF
		Nidovirales (SARS, Coronaviruses)	SARS
		Picornavirales (Foot and Mouth, Coxsackie, Poliovirus)	PICO
		Other ssRNA (+) Strand Viruses, no DNA (Rubella, Hepatitis E, Semliki Forest Virus)	OTssRNA+
	Retro-Transcribing Virus		
		Non-HIV Lentiviruses (EIAV, Caprine Lentiviruses)	LENTI
		Deltaretrovirus (HTLV)	HTLV
		Hepatitis B Virus	HBV
		Other Retroviruses (Mouse Mammary Tumor Virus, Leukemia Viruses, Sarcoma Viruses)	RET
	dsDNA Virus		
		Alphaherpesvirinae (Human Herpesvirus 1/2, Varicellovirus)	AHERP
		Betaherpesvirinae (CMV, Human Herpesvirus 5, Roseolovirus, Muromegalovirus)	BHERP
		Gammaherpesvirinae (Epstein-Barr Virus, Rhadinovirus, Human Herpesvirus 4)	GHERP
		Papillomaviridae (Human papillomavirus)	HPV
		Adenoviruses	ADE
		Polyomaviridae (Simian Vacuolating Virus)	SV40
		Poxviridae (Vaccinia, Pox)	POX
		Other dsDNA virus, No RNA Stage (Caudovirales, African Swine Fever Virus, Baculovirus)	OTdsDNA
	Other Virus	Other Viruses (ssDNA Viruses, dsRNA Viruses, unclassified phages)	OTV
	Actinobacteria/Proteobacteria		
		Mycobacterium (Tuberculosis, Leprosy)	MYCO
		Enterobacteriaceae (E. coli, Salmonella, Yersinia, Shigella, Proteus)	ENT
		Vibrio (V. cholerae, Other Vibrios)	VIB

		Other Gammaproteobacteria (Pseudomonas, Haemophilus)	OTGAM
		Alphaproteobacteria (Rhizobiales, Ricksettias, Anaplasmas)	ALPHA
		Betaproteobacteria (Neisseria, Bordetella)	BETA
	Firmicutes/Other Bacteria		
		Staphylococcus	STAPH
		Listeria	LIST
		Streptococcus	STREP
		Other Bacilli (Anthracis, Cereus, Geobacillus, Enterococcus)	OTBAC
		Clostridiales (Botulinum)	CLOS
		Spirochaetes (Borrelia, Treponema, Leptospiracaeae)	SPIRO
		Chlamydiales (Chlamydia)	CHLA
		Other Bacteria (Boronia, Porphyromonas, Mycoplasmas, Campylobacter)	OTB
	Eukaryota		
		Fungi	FUN
		Plasmodium (P. falciparum, P. vinckeia, P. yoelli)	PLASMO
		Trypanosomatidae (Trypanosoma, Leishmania)	TRYP
		Other Bilateria (Eukaryotic Invertebrates, Nematoda, Platyhelminthes, Shistosoma, Worms, Parasites, Prions)	WORM
		Other Eukaryotes (Entamoebidae, Babesia, Parabasalidea, Coccidia, Theileria, Toxins, Plants)	OTEU

Infectious Disease references are categorized according to the National Center for Biotechnology (NCBI) taxonomy.

There are eight Level 2 categories and 46 Level 3 categories.

Categorization of Transplantation references

**Level 1**	**Level 3**	**Abbreviation**
Transplantation		
	Galactose	GAL
	Graft vs Host Disease	GVH
	H-Y Protein	HY
	Major Histocompatibility Complex	MHC
	Minor Antigens	MINOR
	Allo-Peptides	PEP
	Thrombocytopenia	THRO
	Xenoantigen	XENO
	Blood Groups or Types	BLOOD
	Other (Undefined Transplant/Alloantigen Subtype)	OTH

Transplantation references may be assigned to one of 10 Level 3 categories.

Categorization of Cancer references

Level 1	Level 3	Abbreviation
Cancer		
	Lewis (LeX or LeY)	LEWIS
	Tn, Thomsen-Friedenreich, KH-1, TF	TN
	MUC/Mucin	MUCIN
	Her-2, hTERT	HER2
	Carcinoembryonic Antigen	CEA
	PAP, PSA, PSM, Prostate Cancer	PROS
	MAGE, RAGE, GAGE, PRAME	MAGE
	P53	P53
	RAS	RAS
	Wilm's Tumor Gene, WT1	WT1
	Tyrosinase, TRP2, GP100, TRP1, MART1, SOX10 (Melanoma Associated Antigen)	MAA
	Fetoprotein, VEGF, CTA, SSx2, EphA2, MDM, EGFR, Hepatocellular Carcinoma, Hapten, Recoverin, Survivin	MISC
	CML, AML, Leukemia, Myeloma, BCR-ABL, Lymphoma	LEU
	NY-ESO	NYESO
	HERT (hTERT, Telomerase)	TERT
	Multiple Subtypes/Multipeptide Vaccines	MULTI
	Glioma/SART	GLIO
	Renal (RCC)	RENAL
	P815	P815
	Other (Undefined Subtype)	OTH

Cancer references may be assigned to one of 20 Level 3 categories.

Categorization of Other references

**Level 1**	**Level 2**	**Level 3**	**Abbreviation**
Other			
	Non-Peptidic Antigen		
		DNP, DNFB, TNP, TNCB	DNP
		Other Haptens	OTAP
		Galactose (Sugars)	GAL
		Gangliosides	GANG
	Model Antigen		
		Class II-associated Invariant Chain Peptides	CLIP
		Lysozyme (HEL)	LYS
		Myoglobin	MYO
		Pigeon Cytochrome C, Other Cytochromes	PCYT
		Analog, Antagonist	ANA
	Other		
		Monoclonal Antibodies	MOAB
		B Cell Other	BOT
		T Cell Other	TOT
		Peptide Motifs	MO
		Structure	ST
		Naturally Processed Peptides	NP

Other references contain epitope information that is not described by the six well-defined classes. There are three Level 2 categories and 15 Level 3 categories.
