# Supplementary material for: Cost sensitive hierarchical document classification to triage PubMed abstracts for manual curation
Source: BMC Bioinformatics. 2011 Dec 19;12:482. doi: 10.1186/1471-2105-12-482 (PMC3314711; doi:10.1186/1471-2105-12-482)
Supplement: Additional file 2 — Level 1 uniform cost and cost sensitivity matrices. The uniform cost and cost sensitivity matrices at Level 1. [file 1471-2105-12-482-S2.PDF]

Confusion matrix with uniform cost	Confusion matrix with cost sensitivity

Confusion matrix with uniform cost (rows: **Human Expert**; columns: **Classifier**)

	a	b	c	d	e	f	g
a	**0**	1	1	1	1	1	1
b	1	**0**	1	1	1	1	1
c	1	1	**0**	1	1	1	1
d	1	1	1	**0**	1	1	1
e	1	1	1	1	**0**	1	1
f	1	1	1	1	1	**0**	1
g	1	1	1	1	1	1	**0**

Confusion matrix with cost sensitivity (rows: **Human Expert**; columns: **Classifier**)

	a	b	c	d	e	f	g
a	**0**	1	1	1	5	5	5
b	1	**0**	1	1	5	5	5
c	1	1	**0**	1	5	5	5
d	1	1	1	**0**	5	5	5
e	1	1	1	1	**0**	0.2	0.2
f	1	1	1	1	0.2	**0**	0.2
g	1	1	1	1	0.2	0.2	**0**

Level 1 Category Key: a) Allergy b) Autoimmunity c) Infectious Disease d) Transplantation e) HIV f) Cancer g) Other

Additional file 2
