# Supplementary material for: Cost sensitive hierarchical document classification to triage PubMed abstracts for manual curation
Source: BMC Bioinformatics. 2011 Dec 19;12:482. doi: 10.1186/1471-2105-12-482 (PMC3314711; doi:10.1186/1471-2105-12-482)
Supplement: Additional file 3 — Diabetes Level 3 uniform and cost sensitivity matrices. Uniform cost and cost sensitivity values for Diabetes references at Level 3. Cost sensitivity was implemented when Other was present as a category. Columns represent predictions by the classifier and rows represent the Level 3 category assigned by a human expert. The last row ("Total Incorrect") represents the number of references in which the classifier's prediction was overruled by the human expert. With the implementation of cost sensitivity, fewer references were predicted into the Other category. [file 1471-2105-12-482-S3.PDF]

| Confusion matrix with uniform cost | Confusion matrix with cost sensitivity |
|---|---|

**Confusion matrix with uniform cost** (rows: Human Expert; columns: Classifier)

| | a | b | c | d | e | f | g |
|---|---|---|---|---|---|---|---|
| a | **0** | 1 | 1 | 1 | 1 | 1 | 1 |
| b | 1 | **0** | 1 | 1 | 1 | 1 | 1 |
| c | 1 | 1 | **0** | 1 | 1 | 1 | 1 |
| d | 1 | 1 | 1 | **0** | 1 | 1 | 1 |
| e | 1 | 1 | 1 | 1 | **0** | 1 | 1 |
| f | 1 | 1 | 1 | 1 | 1 | **0** | 1 |
| g | 1 | 1 | 1 | 1 | 1 | 1 | **0** |

**Confusion matrix with cost sensitivity** (rows: Human Expert; columns: Classifier)

| | a | b | c | d | e | f | g |
|---|---|---|---|---|---|---|---|
| a | **0** | 1 | 1 | 1 | 1 | 5 | 1 |
| b | 1 | **0** | 1 | 1 | 1 | 5 | 1 |
| c | 1 | 1 | **0** | 1 | 1 | 5 | 1 |
| d | 1 | 1 | 1 | **0** | 1 | 5 | 1 |
| e | 1 | 1 | 1 | 1 | **0** | 5 | 1 |
| f | 0.2 | 0.2 | 0.2 | 0.2 | 0.2 | **0** | 0.2 |
| g | 1 | 1 | 1 | 1 | 1 | 5 | **0** |

**Diabetes Level 3 Category Key**: a) GAD b) HSP c) IA2 d) IGRP e) INSULIN f) OTH g) VAR

**Classifier with uniform cost** (rows: Human Expert)

| | GAD | HSP | IA2 | IGRP | INSULIN | OTH | VAR |
|---|---|---|---|---|---|---|---|
| GAD | **129** | 0 | 0 | 1 | 2 | 11 | 5 |
| HSP | 0 | **17** | 0 | 0 | 1 | 1 | 0 |
| IA2 | 1 | 0 | **21** | 0 | 0 | 2 | 2 |
| IGRP | 1 | 0 | 0 | **12** | 0 | 2 | 1 |
| INSULIN | 1 | 0 | 0 | 1 | **110** | 9 | 5 |
| OTH | 11 | 1 | 3 | 3 | 14 | **42** | 5 |
| VAR | 11 | 0 | 2 | 2 | 5 | 5 | **4** |
| **Total Incorrect: 108** | 25 | 1 | 5 | 7 | 22 | 30 | 18 |

**Classifier with cost sensitivity** (rows: Human Expert)

| | GAD | HSP | IA2 | IGRP | INSULIN | OTH | VAR |
|---|---|---|---|---|---|---|---|
| GAD | **140** | 0 | 1 | 1 | 3 | 0 | 3 |
| HSP | 1 | **18** | 0 | 0 | 0 | 0 | 0 |
| IA2 | 2 | 0 | **21** | 0 | 0 | 0 | 3 |
| IGRP | 3 | 0 | 0 | **13** | 0 | 0 | 0 |
| INSULIN | 3 | 0 | 0 | 1 | **120** | 1 | 1 |
| OTH | 26 | 1 | 3 | 5 | 31 | **9** | 4 |
| VAR | 16 | 0 | 3 | 2 | 4 | 1 | **3** |
| **Total Incorrect: 119** | 51 | 1 | 7 | 9 | 38 | 2 | 11 |
